# Infectious keratitis: an update on epidemiology, causative microorganisms, risk factors, and antimicrobial resistance

**DOI:** 10.1038/s41433-020-01339-3

**Published:** 2021-01-07

**Authors:** Darren Shu Jeng Ting, Charlotte Shan Ho, Rashmi Deshmukh, Dalia G. Said, Harminder S. Dua

**Affiliations:** 1 Academic Ophthalmology, Division of Clinical Neuroscience, School of Medicine, University of Nottingham, Nottingham, UK; 2 Department of Ophthalmology, Queen’s Medical Centre, Nottingham, UK

**Keywords:** Corneal diseases, Epidemiology, Risk factors

## Abstract

Corneal opacity is the 5th leading cause of blindness and visual impairment globally, affecting ~6 million of the world population. In addition, it is responsible for 1.5–2.0 million new cases of monocular blindness per year, highlighting an ongoing uncurbed burden on human health. Among all aetiologies such as infection, trauma, inflammation, degeneration and nutritional deficiency, infectious keratitis (IK) represents the leading cause of corneal blindness in both developed and developing countries, with an estimated incidence ranging from 2.5 to 799 per 100,000 population-year. IK can be caused by a wide range of microorganisms, including bacteria, fungi, virus, parasites and polymicrobial infection. Subject to the geographical and temporal variations, bacteria and fungi have been shown to be the most common causative microorganisms for corneal infection. Although viral and *Acanthamoeba* keratitis are less common, they represent important causes for corneal blindness in the developed countries. Contact lens wear, trauma, ocular surface diseases, lid diseases, and post-ocular surgery have been shown to be the major risk factors for IK. Broad-spectrum topical antimicrobial treatment is the current mainstay of treatment for IK, though its effectiveness is being challenged by the emergence of antimicrobial resistance, including multidrug resistance, in some parts of the world. In this review, we aim to provide an updated review on IK, encompassing the epidemiology, causative microorganisms, major risk factors and the impact of antimicrobial resistance.

## 摘要

角膜混浊是全球致盲和视力障碍的第五大原因, 世界范围内大约600万人受其影响。此外, 角膜混浊每年导致的单眼失明约150-200万例, 突显其对人类健康造成的持久性负担。在感染、创伤、炎症、变性和营养缺乏等所有的致病因素中, 感染性角膜炎 (IK) 是发达国家和发展中国家角膜病致盲的主要原因, 大概每100000人口中2.5-799人罹患此病。IK可由多种微生物引起, 包括细菌、真菌、病毒、寄生虫和多重感染。受地理和时间变化的影响, 细菌和真菌已被证明是角膜感染最常见的病原微生物。虽然病毒性角膜炎和棘阿米巴角膜炎并不常见, 但在发达国家, 它们是角膜病致盲的重要原因。接触镜的佩戴、外伤、眼表疾病、眼睑疾病以及眼部手术已证实是IK的主要危险因素。广谱抗生素是目前IK治疗的主要选择, 但是在世界某些地区, 其有效性正在受到抗菌药物耐药性的挑战, 其中包含多重耐药性等。本篇综述旨在提供有关IK的最新进展, 包括流行病学、病原微生物、主要危险因素以及抗生素耐药性对于疗效的影响。

## Introduction

Corneal opacity represents the 5th leading cause of blindness globally, accounting for ~3.2% of all cases [1]. The recent World Health Organisation (WHO) report highlighted that ~6 million of the world population are affected by cornea-related blindness or moderate/severe visual impairment, including 2 million of those who are affected by trachoma [1, 2]. In addition, corneal opacity is estimated to be responsible for 1.5–2.0 million cases of unilateral blindness annually, highlighting an ongoing unchecked burden on human health [3, 4].

Any significant insult to the cornea such as infection, trauma, inflammation, degeneration, or nutritional deficiency can result in corneal opacity with visual impairment. Among all, infectious keratitis (IK) has been shown to be the most common cause for corneal blindness in both developed and developing countries [5]. According to a nationwide study, IK was shown to be the most common cause of all corneal blindness in China, primarily attributed to increased risk of trauma, low socioeconomic status and illiteracy [6]. IK is a common yet potentially vision-threatening ophthalmic condition, characterised by acute ocular pain, decreased vision, corneal ulceration, and/or stromal infiltrates [5]. Previously, it has been recognised as a “silent epidemic” in the developing world [3], and recently, a consortium-led proposal has suggested the designation of IK as a “neglected tropical disease (NTD)” [7], adding on to the list of NTDs in ophthalmology (i.e. trachoma, onchocerciasis and leprosy). The proposal to attain status of an NTD aims to draw concerted global effort to tackle IK in under-resourced tropical countries, to ameliorate the societal and humanistic burden of IK.

IK can be caused by a wide variety of pathogens including bacteria, fungi, protozoa and viruses. In addition, polymicrobial infection has shown to be accountable for ~2–15% of all IK cases [8–11]. As the ocular surface is equipped with highly regulated innate and adaptive defense mechanisms [12], IK rarely occurs in the absence of predisposing factors such as contact lens (CL) wear, trauma, ocular surface diseases (OSDs), and post-corneal surgery, which are some of the common risk factors implicated in IK [13].

IK not only causes visual impairment, but also negatively impacts on the quality of life (QOL) of the affected individuals. A study from Uganda reported that IK affected both vision-related QOL (attributed to vision loss) and health-related QOL (attributed to pain in the acute phase) [14]. The psychological impact on these patients was related to the fear of losing the eye and the social stigma attached. Even when the visual recovery was complete, the individuals affected by IK displayed a lower QOL score than the unaffected controls [14]. Apart from the impact on the individuals which can affect their economic productivity, IK is also responsible for a huge economic burden on society. According to a report in 2010, the US spent an estimated 175 million dollars on the treatment of IK [15]. Furthermore, complications of IK such as corneal perforations and scarring form the major indications of corneal transplants in developing countries such as India, Thailand and China [13], placing additional burden on the limited pool of donor corneas.

Considering that most parts of the world affected by IK are under-resourced, it is highly likely that the actual burden of IK is underestimated due to the lack of surveillance and under-reporting. In view of the global burden of IK, this review aims to provide an updated and comprehensive overview of the epidemiology, causative microorganisms, risk factors and the impact of antimicrobial resistance in relation to IK.

## Epidemiology

### B.1. Incidence

To date, there are limited studies available in the literature that examined the incidence of IK and the majority of studies were conducted more than a decade ago [5]. Depending on the geographical location and study design, the incidence of IK has been estimated to be in the range of 2.5–799 cases per 100,000 population/year [16, 17], particularly more prevalent in the low-income countries. Previous IK studies reported an estimated incidence of 2.5–27.6 per 100,000 population-year in the US [16, 18] and 2.6–40.3 per 100,000 population-year in the UK [19, 20]. Our recent Nottingham IK Study concurred with the findings of these older studies. We observed a relatively stable incidence of 34.7 per 100,000 population-year in Nottingham, UK, between 2007 and 2019 [8], highlighting a persistent burden of IK in the developed countries. Another recent study conducted in Australia similarly demonstrated a low IK incidence of 6.6 per 100,000 population-year during the period of 2005–2015 [21]. However, it is noteworthy that the incidence reported in these two studies is likely to be underestimated as the numbers were based on IK patients who underwent corneal scraping.

In contrast, a substantially higher rate of IK has been reported in under-resourced countries such as South India (113 per 100,000 population-year) [22] and Nepal (799 per 100,000 population-year) [17]. The higher incidence observed in these regions was primarily attributable to the poorer environmental and personal hygiene, lower level of education, agricultural industry, increased risk to work-related corneal trauma and poorer access to sanitation and healthcare facility.

### B.2. Age

The epidemiological patterns and risk factors have been found to vary with demographic factors such as age, gender and socioeconomic status. A tabulated summary of the demographic factors and microbiological profiles of IK is provided in Table 1 [8–10, 13, 21, 23–43].

**Table 1:** Summary of the demographic factors and microbiological profiles of infectious keratitis in the literature published between 2010 and 2020, categorised into six distinct regions. Only studies that reported more than 500 cases are included.

Year	Authors	Study period	Region	Total CS	Age (years)	Female (%)	Positive culture (%)	Organisms^a^			Microbiological profiles^b^
								B (%)	F (%)	A (%)	
*UK and Europe*											
2013	Kaye et al. [23]	1995–2010	Liverpool, UK	2418	–	–	35.7	100	0	0	CoNS (26.3); *Enterobacteriaceae* (15.3); *Streptococci* (13.9)
2017	Tan et al. [9]	2004–2015	Manchester, UK	4229	45.9	–	32.6	90.6	7.1	2.3	CoNS (24.4); *S. aureus* (15.1); *Streptococci* (13.3)
2018	Ting et al. [10]	2008–2017	Sunderland, UK	914	55.9 ± 21.0	52.1	46.1	91.0	4.2	4.8	CoNS (25.9); *S. aureus* (13.6); Streptococci (12.1)
2019	Tavassoli et al. [24]	2006–2017	Bristol and Bath, UK	2614	47.7 ± 21.2	51.1	38.1	91.6	6.9	1.4	CoNS (36.0); *Pseudomonas* (15.8); *Streptococci* (7.0)
2020	Ting et al. [8]	2007–2019	Nottingham, UK	1333	49.9 ± 22.2	49.6	37.7	92.8	3.0	4.2	*Pseudomonas* (23.6); *S. aureus* (15.9); *Streptococci (*13.5)
*North America*											
2017	Tam et al. [25]	2000–2015	Toronto, Canada	2330	41.6 ± 24.0	53	57.3	86.0	4.9	2.2	CoNS (37); *P aeruginosa* (10); *Streptococcus spp*. (15)
2018	Peng et al. [26]	1996–2015	San Francisco, US	2203	–	–	23.7	100	0	0	*S. aureus* (20.1); *S. viridans* (13.2); *Pseudomonas* (10.9)
2019	Kowalski et al. [27]^c^	1993–2018	Pittsburgh, US	1387	–	–	100	72.1	6.7	5.2	*S. aureus* (20.3); *Pseudomonas* (18.0); *Streptococci*. (8.5)
2020	Asbell et al. [28]^d^	2009–2018	US	6091	–	46.8	100	100	0	0	*S. aureus* (35.9); CoNS (29); *H. influenza* (13)
*South America*											
2011	Cariello et al. [29]	1975–2007	Brazil	6804	42.1 ± 21.4	40	48.6	78.9	11.0	3.6	CoNS (41.2); *S. aureus* (33.1); *Pseudomonas* (18.5)
2013	Marujo et al. [30]	2005–2009	Brazil	2049	45	45	71.6	80.3	7.0	6	*Staphylococci* (52.5); *Corynebacterium* (14.3); *Streptococci* (10.1)
2015	Hernandez-Camarena et al. [31]	2002–2011	Mexico	1638	45	51.4	38.0	88	12	0	*S. epidermis* (27.4); *Pseudomonas* (12.1); *S. aureus* (9.0)
2016	Yu et al. [32]	1975–2010	Brazil	859	–	42.1	40.3	100	0	0	CoNS (23.8); *S. aureus* (20.9); *Pseudomonas* (14.2)
*Asia*											
2011	Rautaraya et al. [33]	2006–2009	India	997	–	29.9	74.6	23.4	26.4	1.4	*Aspergillus spp*. (23.1); *Fusarium spp*. (19.2); *Staphylococci* (5.4)
2012	Lin et al. [34]	2006–2009	India	5221	–	–	58	35.7	63.0	1.3	*Fusarium spp*. (15.5); *S. pneumoniae* (7.3); *Pseudomonas* (5.0)
2013	Kaliamurthy et al. [35]	2005–2012	India	2170	45.7 ± 16.6	41.3	77	37.2	22.7	1.0	*S. epidermis* (44.0); *S. aureus* (19.5); *S. pneumonia* (11.6)
2015	Lalitha et al. [36]	2002–2012	India	23,897	–	–	59	24.7	34.3	2.2	*Fusarium spp*. (14.5); *Aspergillus spp*. (8.8); *S. pneumoniae* (7)
2015	Wang et al. [37]	2013–2014	China	1000	–	31.8	53.5	0	100	0	*Aspergillus spp*. (53.8); *Fusarium spp*. (19.3)
2016	Hsiao et al. [38]	2003–2012	Taiwan	2012	–	–	49.3	81.1	16	1.1	*Pseudomonas* (24.4); CoNS (16.6); *Propionibacterium* (9.1)
2017	Zhang et al. [39]	2006–2015	China	6220	45.3 ± 22.1	40.6	18.2	100	0	0	*S. epidermis* (29.3); *P. aeruginosa* (11);
2018	Khor et al. [13]	2012–2014	Asia	6626	46.0	39.2	70.7	38	32.7	–	*Fusarium spp*. (18.3); *Pseudomonas* (10.7); *Aspergillus flavus* (8.3)
2019	Acharya et al. [40]	2015–2017	India	1169	–	–	100	100	0	0	CoNS (46.3); *Pseudomonas spp*. (16.2); *Streptococci* (15.5)
2019	Lin et al. [41]	2010–2018	China	7229	–	–	42.8	52.7	57.6	0	CoNS (28.6); *Fusarium spp*. (23.5); *Aspergillus spp*. (12.2)
*Africa and Middle East*											
2016	Politis et al. [42]	2002–2014	Israel	943	47.0 ± 25.2	47	47.9	91.8	8.2	0	CoNS (32.8); Pseudomonas (19.3); S. pneumonia (13.0)
*Australasia*											
2019	Cabrera-Aguas et al. [43]	2012–2016	Sydney, Australia	1084	54	48	66	100	0	0	CoNS (45.8); *Pseudomonas spp*. (12.2); *S. aureus* (11.7)
2019	Green et al. [21]	2005–2015	Queensland, Australia	3182	53 ± 22.6	47.6	73.6	93.1	6.3	0.6	CoNS (33.9); *Pseudomonas spp*. (17.7); *S. aureus* (11.2)

*CS* corneal scrapes, *CoNS* coagulase negative staphylococci.

^a^Breakdown of organisms; B = Bacteria, F = Fungi, A = Acanthamoeba.

^b^The three most common microorganisms isolated in the study.

^c^Included all types of ocular infection.

^d^Included all types of ocular infection but restricted to bacterial infection only.

IK has been shown to affect individuals across all age groups. Based on large-scale studies (>500 patients), IK most commonly affected people aged between 30 and 55 years (Table 1) [8–10, 13, 21, 24, 25, 29, 31, 35, 37, 39, 42, 43], primarily attributed to the underlying risk factors such as CL wear and ocular trauma associated with the working age group. Patients affected by trauma-related IK secondary to agricultural products and foreign bodies are usually around 45–55 years old [18, 44]. The employed workforce of some developing countries is mainly composed of farmers and manual labourers, rendering them more susceptible to IK of traumatic aetiology [13, 45]. On the other hand, patients affected by CL-related IK are usually between 25 and 40 years old [18, 44, 46, 47].

Although prevalence of IK is generally low in the extremes of age [18, 48–51], IK may serve as a major contributor to childhood blindness in some countries. For instance, IK was shown to be the second most common cause of visual impairment in children aged <15 years in Uganda [52]. Ophthalmia neonatorum, defined as conjunctivitis occurring in newborns within 28 days of life, is another important cause of childhood corneal blindness in developing countries, particularly when it is affected by *Neisseria gonorrhoea* where bilateral ocular involvement is common [4].

In addition, some studies have demonstrated that elderly patients affected by IK were associated with poor visual outcome (around 40–75% with visual acuity of <6/60) and higher rate of complications such as corneal melting, perforation and loss of eye (i.e. evisceration or enucleation) [11, 53, 54]. This might be related to the higher rate of ocular co-morbidities and the delay in presentation and/or diagnosis of IK as elderly patients are usually dependent on spouse or family when seeking medical care and they may relate their condition to “normal” age-related changes [55, 56].

### B.3. Gender

The majority of studies did not observe any gender predilection in IK (Table 1). However, when gender difference or predominance exists, it is usually attributed to the underlying risk factors in different regions. For instance, CL-related IK has been shown to exhibit a female predominance of 57–69% [18, 44, 46, 57], whereas trauma-related IK is associated with a male predominance of 74–78% [18, 44, 46], correlating with a high male prevalence (58–75%) of IK in the under-resourced regions such as South America [29, 32], Asia [13, 45, 49, 58], and Africa [51, 59, 60]. Interestingly, a study in Nepal [49] found that there are significantly more male than female patients across all the age groups. This might be due to a combination of higher rate of trauma, lower number of CL wear, and reduced opportunities among the females to access medical services due to cultural customs.

### B.4. Socioeconomic status and level of education

Low socioeconomic status has been shown to increase the risk of developing IK, primarily attributed to poor education, lack of ocular protection and personal hygiene, and limited access to eye care in rural communities [6, 13, 45, 51, 61]. In Asia and Africa, amongst those who were diagnosed with IK, ~45–71% of the patients were illiterate and 62–79% of them resided in rural areas with a poorer access to healthcare facilities [51, 60, 62]. In addition, it was found that farmers, rural residents and illiterates were at a higher risk of refractory IK with poorer outcomes [51].

In some countries such as Nigeria and Malawi, residents in rural communities were shown to be more likely to self-medicate or approach village healers for traditional eye medicine [59, 63]. Although it would be unfair to conclude that all therapies performed by traditional healers are inimical, common beliefs or practises of applying breast milk or plant products directly to the eye may actually worsen their keratitis [63]. In addition, patients who had prior use of traditional eye medicine tended to present later to the eye care professionals, resulting in delayed treatment and poorer visual outcome [63]. Another study conducted in Nepal reported almost half of the patients with keratitis did not use any medication, self-medicated or treated with undocumented medicine [61].

## Causative microorganisms

A wide range of microorganisms, including bacteria, fungi, protozoa (particularly Acanthamoeba), and viruses, are capable of causing IK. Recently, Ung et al [5]. have provided a comprehensive summary of the literature concerning the causative microorganisms of IK (up to June 2018). In view of the recent growing literature, this section aimed to summarise the evidence based on large IK studies (>500 sample size) published during 2010–2020 (Table 1) [8–10, 13, 21, 23–43].

### C.1. Bacteria

Bacteria are commonly categorised into Gram-positive and Gram-negative bacteria based on the difference in the compositions of bacterial cell envelope. In addition to the universal structure of inner/cytoplasmic membrane, Gram-positive bacteria possess a thick outer cell wall, which is composed of layers of peptidoglycan interspersed with teichoic acids and lipotechoic acids, whereas Gram-negative bacteria consist of a thin middle-layer peptidoglycan and an additional outer membrane primarily made of lipopolysaccharide, which has been shown to play an important role in the pathogenesis of infection (including IK) and the contribution to host inflammatory responses [64, 65].

Bacterial keratitis represents the most common type of IK in most regions, including the UK (91–93%) [8–10, 24], North America (86–92%) [25], South America (79–88%) [29–31], Middle East (91.8%) [42], and Australasia (93–100%) [21, 43]. In terms of specific bacterial strains, coagulase negative staphylococci (CoNS), which are a group of common ocular commensal [66], were shown to be the most commonly isolated organisms (24–46%) in about half of the included studies [9, 10, 21, 23–25, 29, 31, 32, 35, 39–43]. Other common bacteria implicated in IK included *S. aureus* (5–36%)*, Streptococci* spp. (7–16%), *Pseudomonas aeruginosa* (5–24%), *Enterobacteriaceae spp*. (15%), *Corynebacterium spp*. (14%), and *Propionibacterium spp*. (9%; see Table 1). Over the past decade, there were several studies in the UK documenting a significant increase in *Moraxella* keratitis, which are often associated with longer corneal healing time [8–10]. Interestingly, *Nocardia* keratitis, a rare cause of IK, was identified as the third most common microorganism (11% of all cases) in the Steroids for Corneal Ulcers Trial (SCUT), and the outcome was found to be negatively influenced by the use of topical steroids [67, 68]. Acid-fast bacilli such as non-tuberculous mycobacteria (NTM) serve as another important group of pathogens that are capable of causing IK [69]. NTM keratitis is commonly associated with refractive surgery and trauma, and it often requires prolonged and aggressive treatment for complete eradication, largely attributed to their propensity to form biofilms [69, 70].

### C.2. Fungi

Fungi can be broadly divided into two categories, namely filamentous and yeast or yeast-like fungi. Filamentous fungi such as *Fusarium spp*. and *Aspergillus spp*. normally thrives in tropical climates whereas yeast-like fungi such as Candida spp. were more commonly observed in temperate regions [71]. Several studies have demonstrated that *Fusarium spp*. (13–24%) and *Aspergillus spp*. (8–30%) were the main causes of IK in Asia, particularly India and China (Table 1) [13, 33, 34, 36, 37, 41]. In 2018, the Asian Cornea Society Infectious Keratitis Study (ACSIKS) included more than 6000 patients from eight Asian countries and re-confirmed the dominance of *Fusarium spp*. keratitis within China (26%) and India (31%) established two decades ago [72–74]. Although the prevalence of fungal keratitis in temperate regions such as the UK, Europe and North America was reportedly lower, the growth of yeast-like fungi such as *Candida spp*. is relatively common in patients with history of corneal transplantation or OSDs [44]. In view of the recent improvement in the diagnostic techniques, rare pathogens such as *Cryptococcus curvatus*, *Arthrographis kalrae*, *Pythium spp*., and many others are increasingly being identified and reported as rare causes of fungal keratitis [75–77].

### C.3. Protozoa

Acanthamoeba is a free-living protozoan that is found ubiquitously in the environment such as water, soil, air and dust [78]. Although not as common as bacterial or fungal keratitis, *Acanthamoeba* keratitis serves as another important cause of IK as it is often associated with prolonged treatment course and poor visual outcome [78]. It was estimated that *Acanthamoeba* keratitis affects 1–33 per million CL wearers per year [78]. In the UK, Carnt et al [79]. recently confirmed an outbreak of *Acanthamoeba* keratitis in the South East England during 2010–2016, with an approximately threefold increase compared to the preceding decade.

Based on recent large studies, *Acanthamoeba* keratitis accounts for ~0–5% of all IK (Table 1). Most of the *Acanthamoeba* keratitis were observed in CL wearer (71–91%) [32, 60, 80]. However, non-CL wearers can also develop this infection if their eyes are exposed to contaminated water, soil or dust, [81, 82]. One of the Indian studies reported that only 4% of *Acanthamoeba* keratitis cases were associated with CL wear and the remainder were associated with trauma and/or exposure to contaminated water [82]. In addition, the clinical features of non-CL related *Acanthamoeba* keratitis may differ from CL-related cases [82]. Moreover, *Acanthamoeba* sclerokeratitis may manifest as a rare but difficult-to-treat clinical entity that is usually associated with poor clinical outcomes [83].

Microsporidial keratitis represents another type of parasitic IK that accounts for ~0.4% cases of all IK [84]. It is mainly observed in Asian countries and may manifest as superficial keratoconjunctivitis or stromal keratitis. It is commonly associated with ocular trauma, exposure to contaminated water/soil, and potentially acquired immunodeficiency syndrome [84, 85].

### C.4. Viruses

Viral keratitis, most commonly in the form of herpes simplex keratitis (HSK) and herpes zoster keratitis (HZK), represents a common cause of IK [86, 87]. However, as viral keratitis cases are commonly treated based on their typical clinical appearance (e.g. dendritic corneal ulcer in HSK) and/or previous ocular history, the majority of cases did not require any microbiological investigation and hence were not captured in many IK studies. Nonetheless, the ACSIKS study demonstrated that viral keratitis represented the most common cause (46%) of IK in China, primarily attributed to HSK (24%) and HZK (17%) [13]. Another two studies, conducted in Egypt and China, respectively, observed that 15–21% of IK were caused by herpetic keratitis [51, 58]. Based on these results, it is likely that viral keratitis represents an important and common cause of IK in many other regions, though further studies are required to elucidate this. Herpetic keratitis is often associated with neurotrophic keratopathy, which can result in poor corneal healing, increased risk of further IK and other corneal complications such as melting and perforation [86, 88].

### C.5. Polymicrobial infection

Polymicrobial keratitis (IK caused by two or more causative microorganisms) has been reported in around 2–15% of all IK cases [8–11, 21]. Depending on the study design and the definition used, polymicrobial keratitis may include two or more types of organisms from the same category (e.g. bacteria-bacteria, fungus-fungus) or different categories (bacteria-fungus, fungus-protozoan). Polymicrobial keratitis often poses significant diagnostic and therapeutic challenges, and usually fares worse than monomicrobial keratitis [11, 75, 89]. Khoo et al [11]. observed that patients affected by polymicrobial keratitis (median of 6/60 vision) had a significantly worse visual outcome as compared to those affected by bacterial keratitis (median of 6/18 vision) or culture negative IK (median of 6/9 vision). In another retrospective comparative study, Lim et al [89]. demonstrated that medical therapy was sufficient to resolve all monomicrobial IK cases but only 81% of polymicrobial IK. In view of the relatively common occurrence of polymicrobial keratitis and variably low culture yield of current microbiological investigation, clinicians should always maintain a low threshold of repeating corneal scraping if patients are not responding to either antibacterial or antifungal therapy, even in the presence of positive culture results.

### C.6. Seasonal variations

Pathogens are tremendously adaptive to climate and seasonality. Many studies have shown that IK was most prevalent during the summer season, with *P. aeruginosa* being one of the most frequently isolated microbes [34, 90, 91]. *P. aeruginosa* is a well-recognised organisms associated with environmental water as in swimming pools [92] and CL [44, 46, 48, 93, 94]. The seasonal predilection of IK during summer is attributed to the likely increased use of CL wear and engagement in water activities. On the other hand, several studies have shown that the incidence of fungal keratitis in India peaked during the windy and harvest seasons, primarily related to a higher risk of trauma secondary to agricultural activities and agricultural debris being blown in the eyes by the wind [34, 62].

Seasonal variation was similarly observed in *Acanthamoeba* keratitis, though with conflicting results. Lin et al [34]. observed that *Acanthamoeba* keratitis occurred more commonly during summer in South India, potentially related to the higher temperature and increased risk of corneal trauma during windy seasons, whereas Walkden et al [91]. reported an increase in *Acanthamoeba* keratitis during the winter in the UK.

## Major risk factors

In the majority of IK cases, local and/or systemic risk factors are usually present. The most common risk factors include CL wear, ocular trauma, OSDs (e.g. dry eye diseases (DEDs), neurotrophic keratopathy, rosacea, etc.), lid diseases, post-corneal surgery (e.g. keratoplasty, corneal cross-linking (CXL)), and systemic diseases (e.g. diabetes, immunosuppression), amongst others. A tabulated summary of large IK studies reporting the risk factors of IK is provided in Table 2 [11, 13, 18, 29, 32, 35, 44–46, 48–51, 58–62, 93–101].

**Table 2:** Summary of risk factors and associated organisms of infectious keratitis in the literature published between 2010 and 2020, categorised into six distinct regions. Only studies that reported more than 200 cases are included.

Year	Authors	Study period	Region	Patients	Age, years (Mean ± SD)	Female, %	Risk factors (%)
*UK and Europe*							
2013	Dethorey et al. [93]	2005–2011	France	268	45	50.4	CL (48.1), OSD (33.7), POS (17.5)
2018	Ferreira et al. [48]	2007–2015	Portugal	235	50.0 ± 20.7	55.1	CL (28.9), trauma (28.9), DM (13)
2020	Sagerfors et al. [95]	2004–2014	Sweden	398	49.5	57	CL (45.5), OSD (9.8), corneal transplant (9.5)
*North America*							
2010	Jeng et al. [18]	1998–1999	US	302	42.8	57.3	CL (55), OSD (19.2), trauma (11.9)
2011	Keay et al. [44]	2001–2007	US	733	47.9	46.8	CL (36.6), OSD (28.5), trauma (24.6)
2013	French et al. [96]^#^	2010	US	2124	39.2	53.5	Scleral ectasia (4.8), CL (4.8), corneal abrasion (3.1)
2015	Truong et al. [97]	2009–2014	US	318	42.9	40.3	CL (41), OSD (28), trauma (17), topical steroid (4)
*South America*							
2011	Cariello et al. [29]	1975–2007	Brazil	16742	42.1 ± 21.4	40	POS (22.4), CL (12.8), trauma (16.4), topical steroid (6.6)
2016	Yu et al. [32]	1975–2010	Brazil	859	–	42.1	Topical medication (30.6), Trauma (24), POS (24), CL (13)
*Asia*							
2011	Kumar et al. [62]	2003–2005	India	200	–	39	Trauma (78.5), OSD (12)
2011	Ganguly et al. [49]	2006–2007	Nepal	1880	–	40.7	Trauma (58), topical steroid (12), OSD (6), CL (5)
2012	Dhakhwa et al. [98]	2007	Nepal	414	–	42.8	Farmers (75.4), trauma (33.3), topical steroid (4.1)
2012	Hussain et al. [99]	2007–2009	Pakistan	228	42.8 ± 21.9	35.1	Trauma (31.5), POS (8.8), topical steroid (6.6)
2012	Deorukhkar et al. [100]	2004–2009	India	852	–	31.7	Trauma (60.2), FB (15.6), POS (9.5)
2013	Kaliamurthy et al. [35]	2005–2012	India	2170	45.7 ± 16.6	41.3	Trauma (64.0), traditional eye medicine (16.9)
2015	Sitoula et al. [101]	2011	Nepal	1644	44 ± 16	42	Trauma (60), dacryocystitis (5)
2016	Pan et al. [58]	2003–2012	China	578	52.4	25.4	Trauma (54.7), URTI (11.9), DM (8)
2018	Khor et al. [13]	2012–2014	Asia	6563	46.0	39.2	Trauma (34.7), CL (10.7), POS (6.8), OSD (4.2)
2018	Chidambaram et al. [45]	2012–2013	India	252	50	36	Trauma (71.8), traditional eye medicine (19.0) topical steroid (9.9), DM (6.7)
2018	Al-Ghafri et al. [50]	2013–2016	Oman	304	52.2 ± 23.2	56.2	Blepharitis (54.3), trachoma (26.0), Other lid diseases (18.1), CL (17.1), Climate droplet keratopathy (15.5)
2018	Gautam et al. [61]	2016	Nepal	259	44.9	54.4	Trauma vegetative material (48), topical steroid (9)
2019	Tong et al. [46]	2012–2016	Singapore	377	33.6 ± 17.2	53.5	CL (64.3), OSD (10), trauma (3.9)
2020	Khor et al. [94]	2010–2016	Malaysia	221	39.5	41.2	Trauma (49.3), CL (23.1), OSD (5.9)
*Africa and Middle East*							
2013	Oladigbolu et al. [59]	1995–2005	Nigeria	228	–	43.4	Trauma (51.3), traditional eye medication (17.1), topical steroid (5.7)
2014	Mandour et al. [51]	2010–2013	Egypt	340	–	41.2	Trauma (50), POS (14.7), topical steroid (11.8)
2018	Zbiba et al. [60]	2011–2016	Tunisia	230	–	40	OSD (58.7), Trauma (51.3), DM (16), topical steroid (10.9), CL (9.5)
*Australasia*							
2020	Khoo et al. [11]	2012–2016	Australia	979	54.7 ± 21.5	48.3	CL (63), topical steroid (24), OSD (18)

*CL* contact lens wear, *POS* previous ocular surgery, *OSD* ocular surface diseases, *FB* foreign bodies, *DM* diabetes, *URTI* upper respiratory tract infection.

^#^The data were based on patients presented to general emergency department; therefore, risk factors might not be accurately documented.

### D.1. Contact lens (CL) wear

CL wear has been recognised as one of the most common risk factors of IK, particularly in developed countries. A study conducted in Northern California reported that the incidence of IK among CL wearers was ~9.3 times higher than the non-CL wearers (130.4 vs. 14.0 per 100,000 person-years) [18]. Based on the large studies (>200 patients) published in the recent literature, CL wear was shown to be the main predisposing factor (29–64%) of IK in developed countries like Portugal [48], France [93], Sweden [95], the US [18, 44, 97], Singapore[46] and Australia [11]. On the contrary, CL-related IK was considerably less common (0–18%) in developing countries due to less number of CL wearers [13, 35, 50, 59, 60], highlighting the geographical disparity in the risk factors as well as the causative microorganisms of IK between high income and low-income countries (Table 2).

The pathogenesis of CL-related IK is complex and multifactorial. Although it is commonly believed that CL-related IK is triggered by superficial injury secondary to CL wear, several studies had refuted this hypothesis as it was shown that the presence or absence of epithelial injury did not influence the risk or severity of IK [65]. Plausible mechanisms of CL-related IK include reduction of tear exchange during blinking (which leads to potential degradation of protective components at ocular surface), tear stagnation under CL (particularly soft CL) resulting in accumulation and adherence of microbes to the cornea, reduced corneal epithelial cell desquamation, and alteration of tear fluid biochemistry [65]. In addition, multiple predisposing factors of CL-related IK have been identified, including the types of CL used (higher risk in soft CL than rigid gas permeable CL), poor CL and CL case hygiene, overnight wear, use of expired CL, types of CL solution used, and CL being prescribed/dispensed by non-ophthalmologists or non-opticians [93, 102–106]. Reports of IK secondary to the use of cosmetic lens and orthokeratology lens have also been highlighted [107, 108].

In terms of underlying aetiologies, CL-related keratitis is most commonly associated with *P. aeruginosa* and *Acanthamoeba spp*., which are both free-living microorganisms that are ubiquitously present in the environment, including water and CL solutions [47]. As noted above, Pseudomonas keratitis is one of the most common causes of IK, especially in the developed countries where there is increased prevalence of CL wear. Yildiz et al [102]. and Tong et al [46]. observed that *P. aeruginosa* was responsible for 63% and 70% of the CL-related IK, respectively. While *Acanthamoeba* keratitis is uncommon, most of these cases (71–91%) were observed in CL wearers [32, 60, 80]. Yu et al [32]. observed that more than 90% of the *Acanthamoeba* keratitis were associated with CL use. In a 32-year Brazilian study of over 6000 IK cases, Cariello et al [29]. reported that CL wearers had a 1.7 times higher risk of developing Acanthamoeba-positive culture than non-CL wearers. Interestingly, CL wear was also shown to be a major risk factor for fungal keratitis in a US study [44].

### D.2. Trauma

Trauma serves as another common risk factor for IK in both developed and developing countries. Based on the IK studies reported in the literature, farmers (54–70%) and manual labour workers (11–17%) constituted the main occupations in Asia [13, 45, 49, 51, 58, 59, 109]. These groups of workers were at a high risk of developing IK due to the increased occupational exposure to plant materials and foreign bodies, which was frequently compounded by the lack of eye protection [45, 51, 58, 98, 109].

Fungal keratitis is by far the most common cause (47–83%) of trauma-related IK, especially in regions such as Asia and Africa which are dominated by agricultural communities [45, 51, 58, 60, 94]. Occupational exposures to vegetative matter, organic materials and animal products, predominantly in males in the working age group, are the main causes in these regions. The risk of fungal keratitis is further magnified by tropical climates, which are conducive to fungal growth [51, 60]. Cariello et al [29]. observed that the risk of developing culture-proven fungal keratitis was increased by four times if the patients suffered from plant-related trauma. In addition, some studies demonstrated that trauma-related IK fared worse than non-traumatic cases [46, 58]. Pan et al [58]. conducted a 10-year study in China and revealed that patients who presented with trauma-related IK were at a high risk of developing fungal keratitis and requiring surgical interventions (89%), including therapeutic keratoplasty and evisceration/enucleation.

On the other hand, the majority of trauma-related IK reported in European countries were caused by Gram-positive bacteria, including CoNS, *S*. aureus, *Streptococci*, and *Corynebacterium* [48, 95]. These are common ocular surface commensals, which have the ability to tolerate hot and dry climates in temperate and sub-tropical zones [51, 110, 111]. Corneal trauma resulting from non-vegetative matter with consequent secondary opportunistic infection with ocular surface commensals could explain the high rate of Gram-positive infection in trauma-related IK in this region.

### D.3. Ocular surface and eyelid diseases

Ocular surface diseases (OSDs), encompassing DEDs, blepharitis, neurotrophic keratopathy, Steven–Johnson syndrome, ocular cicatricial pemphigoid and bullous keratopathy, have been identified as one of the main risk factors for IK in both developed and developing countries [18, 44, 49, 60, 97, 112]. OSD-related IK is most commonly caused by Gram-positive bacteria (around 60–80%) [11, 60, 95, 112], which constitute the main group of ocular surface commensals. In particular, CoNS and *S. aureus* were shown to be the main culprits in OSD-related IK [95, 112].

DED is the most common OSD that is characterised by “*a loss of tear film homeostasis with ocular symptoms, in which tear film instability and hyperosmolarity, ocular surface inflammation and damage, and neurosensory abnormalities play etiological roles*” [113]. The dysregulated ocular surface health can lead to breakdown of the corneal epithelium, a vital ocular surface defence, and ocular surface inflammation, consequently increasing the risk of IK [60, 114].

Posterior blepharitis or meibomian gland disease (MGD) is a common eyelid disease, which is difficult to cure. It can lead to an array of ocular surface complications, including evaporative DED, marginal keratitis and IK, amongst others [115]. Meibomian gland abnormalities (e.g. gland dropout and hyperkeratinisation), alteration of the secreted lipid products, and the dysregulation of bacterial populations and their corresponding lipase or esterase activity are believed to contribute to the ocular surface inflammation and infection. In a 5-year Australian study, MGD was shown to be the most common cause (79%) of OSD implicated in IK [112]. In addition, nasolacrimal duct obstruction (NLDO) can also increase the risk of IK, primarily attributed to tear stagnation and reduction of tear exchange, resulting in the accumulation of microbes and debris on the ocular surface with increased risk of IK. Chidambaram et al [45]. showed that NLDO could increase the risk of fungal and bacterial IK, particularly *S. pneumonia* keratitis.

### D.5. Post-ocular surgery

IK may occur following various ocular surgeries, including corneal transplant, refractive surgery, CXL, pterygium surgery, cataract surgery, and others [29, 51, 116, 117]. Corneal transplant serves as the main sight-restoring surgery for a wide range of corneal diseases, though postoperative complications such as graft failure and IK may develop. In a retrospective study of over 2000 corneal transplants, Dohse et al [116]. reported an incidence of post-keratoplasty IK of 4%, with loose and broken sutures being reported as one of the most common risk factors (24%) [116]. Cariello et al [29]. demonstrated that 22% of the IK cases were associated with prior ocular surgery, particularly corneal graft (56%). In addition, the paradigm shift of penetrating keratoplasty to lamellar keratoplasty has created a new array of host-graft interface complications such as interface infectious keratitis (IIK), which often causes diagnostic and therapeutic challenges due to the deep-seated location of the infection [118, 119]. We have recently highlighted a clinically challenging case of post-endothelial keratoplasty interface fungal keratitis, which required in vivo confocal microscopy for confirmatory diagnosis in the absence of positive culture results [118]. Fortunately the interface infection resolved quickly after the discontinuation of topical steroids and initiation of appropriate antifungal treatment.

Although IK rarely develops after refractive surgery, the significant amount of refractive surgeries performed globally render this an important clinical entity [120]. This was supported by a Brazilian study where refractive surgery was shown to be the second commonest surgery associated with IK [29]. Post-refractive surgery IK is most commonly caused by Gram-positive bacteria and NTM, though fungal and Acanthamoeba infection may also occur [120]. The high rate of Gram-positive bacterial IK following other types of ocular surgeries (e.g. cataract surgery, pterygium surgery) were also observed, most likely as a result of opportunistic infection secondary to ocular surface commensals [51, 93, 95].

In the recent years, CXL has emerged as a therapeutic modality for managing corneal ectactic conditions [121, 122] and moderate-to-severe IK [123–125]. However, the intraoperative removal of corneal epithelium and postoperative insertion of bandage CL (which is the current standard practice in most institutes) can increase the risk of IK following CXL, particularly in patients with OSD such as vernal or atopic keratoconjunctivitis [117, 126, 127]. Post-CXL IK may be further complicated by the reactivation of herpetic keratitis [126] and manifestation of acute hydrops [127] and corneal melt/perforation [117].

### D.6. Use of topical steroids

Steroids are commonly used in ophthalmology as a topical immunosuppressive/immunomodulatory agent to manage a wide range of intraocular and ocular surface inflammatory diseases, including DED, allergic eye disease, non-IK, chemical eye injury, cicatricial conjunctivitis and many others [128, 129]. The recent SCUT study also demonstrated the benefit of adjuvant topical steroids in improving the visual outcome in patients with severe and central bacterial keratitis [67]. In addition to managing OSDs, topical steroids are also frequently used as postoperative topical treatment following intraocular and ocular surface surgeries, including corneal transplantation [130].

However, topical steroids can sometimes act as a double-edge sword. Studies have shown that topical steroids can increase the risk of IK, particularly fungal keratitis and/or polymicrobial keratitis [11, 44, 118]. In a study of 733 fungal keratitis, Keay et al [44]. reported that 13% of the cases were associated with chronic use of topical steroids. In addition, a study has shown that previous use of topical steroid could negatively impact on the clinical outcome of IK, with 73% ending with poor outcome (defined as worse than 6/60 vision, decreased vision during treatment, or perforation) [11]. While topical steroids serve as an effective treatment for stromal HSK, which is primarily an immune-related keratitis [131], its use can potentially exacerbate epithelial HSK and culminate in geographic ulcer [132]. Interestingly, an Indian study showed that 41% of the *Acanthamoeba* keratitis cases were associated with the use of topical steroid [45]. The high rate of prior steroid use might be related to the fact that *Acanthamoeba* keratitis often presents with non-specific corneal epithelial changes and is mismanaged as viral keratitis [104].

### D.7. Systemic immunosuppression

Systemic immunosuppression, either secondary to diseases or immunosuppressive agents, has been shown to increase the risk of IK. Diabetes mellitus serves as one of the most important systemic risk factors for IK. Hyperglycaemia has been shown to facilitate microbial growth and alter the microbiota of ocular surface, including an upregulation of *Pseudomonas spp*. and *Acinetobacter spp*. [133], as well as affect the homeostasis, corneal sensation and wound healing of the corneal epithelium, thereby increasing the risk of IK [134]. Sub-basal corneal nerve plexus of patients with diabetic neuropathy is often affected and can lead to neuropathic keratopathy with complications such as corneal melt and IK [135].

Several large studies have highlighted the association between diabetes and IK (around 8–16%), particularly fungal and bacterial keratitis [45, 58, 60, 136, 137]. Zbiba et al [60]. observed that diabetes was relatively common in patients with bacterial keratitis (15%) and fungal keratitis (16%) as well as mixed bacterial and fungal keratitis (29%). In addition, viral keratitis was also reported to have a high prevalence amongst patients with diabetes [138]. Viruses, particularly HSV, are omnipresent in the general population, with an estimated prevalence of 1.5 per 1000 population [139]. Kaiserman et al [140]. demonstrated that patients with diabetes had a significantly higher incidence and recurrence rate of ocular surface herpetic eye diseases when compared to non-diabetic patients. Pan et al [58]. observed that 17% patients with diabetes had a substantially higher rate of HSK as compared to bacterial or fungal keratitis. Another study described that all patients with diabetes presented with IK were of viral origin, though the sample size was small [51]. The heterogeneity in the subtypes of microorganisms associated with diabetes observed in different studies was likely related to the disparity in the ocular predisposing factors of the studied cohort since more than one risk factor is often present in patients with IK [11].

Apart from diabetes, Jeng et al [18]. observed an approximately tenfold increased risk of IK in individuals affected by human immunodeficiency viruses compared to healthy individuals (238.1 vs. 27.6 per 100,000 population-year), highlighting the importance of host immunity in ocular surface defence. Intriguingly, a study demonstrated that 55% of the patients with HSK had a history of upper respiratory tract infection prior to the infection or recurrence [58]. This could be potentially explained by the mechanism linked to a host cell enzyme called heparanase [141], which is a known contributing factor to the pathogenesis of several viruses, including HSV, respiratory syncytial virus, human papilloma virus, and others. End-stage renal disease, particularly associated with diabetes, was also shown to be a risk factor for IK [142].

## Antimicrobial resistance (AMR)

### E.1. Overview

AMR has been recognised as a major public health crisis in the past two decades, with many infectious organisms developing resistance against previously effective antimicrobial agents [143]. The development of AMR is largely driven by a multitude of factors, including the overuse/abuse of antimicrobial agents in agricultural sectors due to commercial pressure, uncertainty in diagnosis (e.g. bacterial infection vs. viral infection) leading to inappropriate use of antibiotics, financial incentives for prescribing antibiotic, and use of non-prescription antibiotics among the general public, particularly in low- and middle-income countries [143, 144]. From the genetic point of view, bacteria primarily develop AMR through two strategies, namely genetic mutational resistance and horizontal gene transfer. The genetic and mechanistic basis of AMR can be referred to a recent excellent review provided by Munita and Arias [144].

### E.2. AMR in the context of IK

Broad-spectrum topical antibiotic therapy is the gold standard treatment for IK. Depending on the disease severity and clinicians’ preference, antibiotic therapy is commonly administered in the form of dual therapy using cephalosporin and aminoglycoside or monotherapy using fluoroquinolone [145]. As intensive topical antibiotics are applied directly and frequently during the treatment of IK, high concentration of antibiotics can be effectively achieved at the target site (i.e. the infected cornea), which could potentially reduce the risk of AMR in ocular infections. However, a few recent IK studies have highlighted the emergence of AMR in ocular infections, particularly in the US [28], China [41] and India [40]. The driving force is likely to be multifactorial, including the injudicious widespread use of antibiotics in both ocular and systemic infections [146], incorrect dosing regimen [147], and representations of the community prevalence of drug resistance, with consequent colonisation of ocular surface by drug resistant pathogens [148]. For instance, in the SCUT trial, there was a 3.5-fold higher MIC for bacteria isolated from patients who had previous treatment with fluoroquinolones compared to treatment naive patients [149].

A tabulated summary of the literature concerning the in vitro antibiotic susceptibility and resistance of IK-related bacteria is provided in Table 3 [8, 9, 21, 24–26, 28, 31, 35, 38, 40–43, 150]. Overall, fluoroquinolone-resistant, methicillin-resistant and multidrug resistant (MDR; i.e. resistant to 3 or more antibiotics) infections are being increasingly reported in IK [28, 31, 35, 40, 41, 150]. Geographical and temporal factors play a role in the variation of AMR pattern in ocular infections. Reports from Southern India demonstrated that MDR was commonly observed among *S. pneumoniae* (44%), *S. epidermidis* (14.8%), *S. aureus* (14%), and *P. aeruginosa* (6%). However, gatifloxacin—a fourth-generation fluoroquinolone—was effective against the majority of Gram-negative bacteria (~90%), including *P. aeruginosa* and *Acinetobacter spp*., thus its use as a monotherapy in Gram-negative IK was recommended in that region [35]. Another study from Southern China similarly reported an increase in MDR among Gram-positive cocci from 2010 to 2018, while susceptibility to fluoroquinolone and aminoglycoside among Gram-negative bacilli remained stable [41]. In contrast, a Northern India study reported a high rate of resistance of *P. aeruginosa* against ciprofloxacin (57%), moxifloxacin (47%), and aminoglycoside (52–60%) [40], highlighting the geographical disparity in the AMR pattern and the importance of region-specific interrogation of the AMR profile in ocular infections.

**Table 3:** A summary of the in vitro antimicrobial susceptibility and resistance of the causative microorganisms of infectious keratitis.

Year	Authors	Study period	Region	No. of cases	Antibiotic susceptibility (%)^a^		
					CEP	AMG	FQ
*UK and Europe*							
2017	Tan et al. [9]	2004–2015	UK	4229	86 (P); 61 (N);	88 (P); 97 (N)	83 (P); 91 (N)
2019	Tavassoli et al. [24]	2006–2017	UK	2614	–	100 (P); 97.0-100 (N)	91-100 (P); 97-100 (N)
2020	Ting et al. [8]	2007–2019	UK	1333	100 (P); 81 (N)	95 (P); 98-99 (N)	90-100 (P); 98-100 (N)
*North America*							
2017	Tam et al. [25]	2000–2015	Canada	2330	–	96 (P)	96 (P)
2018	Peng et al. [26]	1996–2015	US	2203	–	50-100 (N)	85-100 (P); 80-100 (N)
2020	Asbell et al. [28]	2009–2018	US	6091	–	97 (MSSA); 62 (MRSA); 94 (MS-CoNS); 71 (MR-CoNS); 97 (N)	89-90 (MSSA); 26-29 (MRSA); 88-89 (MS-CoNS); 43-49 (MR-CoNS); 93-100 (N)
*South America*							
2013	Vola et al. [150]	2000–2009	Brazil	566	–	93 (MSSA); 70 (MRSA)	96 (MSSA); 62 (MRSA)
2015	Hernandez-Camarena et al. [31]	2002–2011	Mexico	1638	18-90 (P); 10-92 (N)	42-80 (P); 69-98 (N)	54-100 (P); 87-100 (N)
*Asia*							
2013	Kaliamurthy et al. [35]	2005–2012	India	2170	–	31-95 (P); 90-93 (N)	70.4-98 (P); 74-90 (N)
2016	Hsiao et al. [38]	2003–2012	Taiwan	2012	–	85-88 (N)	89 (P); 94 (N)
2019	Acharya et al. [40]	2015–2017	India	1169	–	73 (P); 89 (N)	69 (P); 69 (N)
2019	Lin et al. [41]	2010–2018	China	7229	84-91 (P); 68-75 (N)	–	63-75 (P); 46-75 (N)
*Africa and Middle East*							
2016	Politis et al. [42]	2002–2014	Jerusalem	943	–	92-94 (P)	97-100% (P)
*Australasia*							
2019	Cabrera-Aguas et al. [43]	2012–2016	Australia	1084	–	86-97 (P); 100 (N)	86-95 (P); 99 (N)
2019	Green et al. [21]	2005–2015	Australia	3182	–	92 (P); 96 (N)	94 (P); 99 (N)

*MSSA* Methicillin-sensitive *Staphylococcus aureus*, *MRSA* Methicillin-resistant *S. aureus*, *MS-CoNS* Methicillin-sensitive coagulase negative staphylococci, *MR-CoNS* Methicillin-resistant coagulase negative staphylococci.

^a^Antibiotic susceptibility is reported for Gram-positive bacteria (P) and Gram-negative bacteria (N) against three common classes of antibiotics, namely cephalosporin (CEP), aminoglycoside (AMG) and fluoroquinolone (FQ).

An increasing trend of MRSA-related ocular infection has also been reported in several studies in the past decade [28, 31, 41]. The Antibiotic Resistance Among Ocular Microorganisms study in the US observed that a high rate of AMR, specifically methicillin resistance, was observed among *Staphylococci spp*. and *Streptococci spp*. and the risk increased with age [28]. More worryingly, ~75% of the MRSA and MR-CoNS were MDR. Another US study demonstrated an increased rate of MRSA-related IK as well as resistance against fluoroquinolones, which questioned their ongoing use as primary monotherapy [26]. Similarly, a 10-year Mexico study showed that 21–79% of the *S. aureus* and 48–71% of the CoNS were resistant to oxacillin (or methicillin). *P. aeruginosa* and other Gram-negative infections displayed resistance against oxacillin (86% and 90%, respectively) and vancomycin (97% and 70%, respectively), with an increasing trend of resistance to ceftazidime observed over time [31]. Another study conducted in Taiwan also highlighted the emerging issue of methicillin resistance, with MRSA accounting for 43% of all Gram-positive IK [38]. On the other hand, an increase in voriconazole resistance was observed in the Mycotic Ulcer Treatment Trial (MUTT)-I for fungal keratitis, with a 2.1-fold increase in the mean MIC per year after adjustment for causative organism [151].

Reassuringly, reports from the UK showed that Gram-positive bacteria exhibited a high susceptibility to cephalosporin (87–100%), but a moderate susceptibility to fluoroquinolone (61–81%). However, Gram-negative bacteria were highly susceptible to both aminoglycoside (97–100%) and fluoroquinolone (91–100%) [8, 9, 24], suggesting that current antibiotic regimen (fluoroquinolone monotherapy or cephalosporin-aminoglycoside dual therapy) could safely remain as the first-line treatment in the UK. In our recent 12-year Nottingham IK Study, we observed an increasing trend of resistance against penicillin over time in both Gram-positive and Gram-negative isolates but a generally good susceptibility to aminoglycosides and fluoroquinolones was maintained; therefore, no change of antibiotic regimen was required [8].

### E.3. Clinical impact

AMR represents a global challenge with a huge impact on morbidity and mortality. It was estimated that 2 million people/year in USA are infected with antimicrobial resistant organisms, with a $20 billion cost incurred on the healthcare system. A recent UK report also predicted a global loss of $100 trillion by 2050 related to AMR [152].

Within the context of IK, AMR was found to negatively affect the clinical outcome of IK. Kaye et al [153]. observed that the corneal healing time of IK was prolonged with the increase of minimum inhibitory concentration (MIC; i.e. antibiotic resistance) of the causative organisms, including *P. aeruginosa*, *S. aureus* and *Enterobacteriaceae spp*., against fluoroquinolone monotherapy. In addition, Lalitha et al [154]. demonstrated that higher level of MIC was associated with a significantly increase risk of corneal perforation in fungal keratitis.

AMR is continuing to increase in an alarming way. There is a pressing need to increase the awareness amongst prescribers on judicious use of antimicrobials, to tighten the control of ‘over the counter (OTC)” antimicrobials in many countries, and to develop novel therapeutic modalities and strategies for IK, including therapeutic CXL and host defence peptides (or previously known as antimicrobial peptides), which hold great promises as a new class of antimicrobials in the future [123, 155–157].

## Conclusions

IK represents a persistent burden on human health in both developed and developing countries. As the incidence of IK is likely to be underestimated in the recent studies, well-designed prospective studies including all types of microorganisms (i.e. bacteria, fungi, protozoa and viruses) are required to truly ascertain the incidence and impact of IK. Understanding of the major risk factors for IK in different regions, particularly CL wear, trauma, OSD, and post-ocular surgery, will facilitate a more effective public health intervention to modify and reduce the risk of IK. The increase rate of AMR in ocular infection in several countries, including the US, China, and India, over the past decade highlights the need for judicious use of antimicrobials, tighter control of OTC antimicrobials and development of new antimicrobials and strategies for therapy. Improvement in the diagnostic yield of microbiological investigations of IK with emerging technologies such as next-generation sequencing and artificial intelligence-assisted platforms could also provide a better guidance on the appropriate use of antimicrobial therapy in the future, ultimately reducing the risk of AMR [158, 159].

## Methods of literature review

Two authors (DSJT and CSH) searched the PubMed (January 1980–May 2020) for relevant articles related to IK. Keywords such as “corneal infection”, “corneal ulcer”, “IK”, “microbial keratitis”, “incidence”, “prevalence”, “epidemiology”, “risk factors”, “antibiotic resistance” and “antimicrobial resistance” were used. There was no restriction to the language used. Bibliographies of included articles were manually screened to identify further relevant studies. The final search was updated on 15 June 2020.

**Fig. 1:**
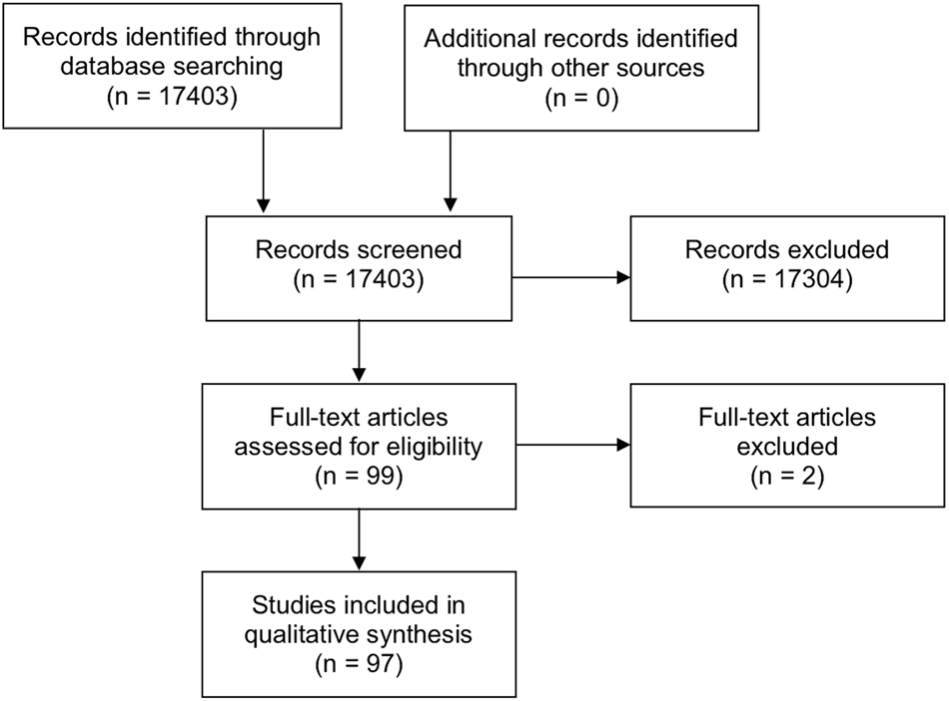
The PRISMA flow chart detailing the process and results of literature search for articles related to infectious keratitis.

A web application designed for systematic reviews, Rayyan (Qatar), was used to help collate the potential studies and expedite the initial screening of abstracts and titles [160]. The titles and abstracts obtained from the searches were independently screened by two authors (DSJT and CSH) to include studies that fulfilled the eligibility criteria. The authors then independently assessed the full-text version of all selected articles and extracted data onto a standardised data collection form for data synthesis. The extracted data included the authors, year of publication, country, sample size, demographic factors, culture results, risk factors and in vitro antibiotic susceptibility. Discrepancies were resolved by group consensus and independent adjudication (HSD) if consensus could not be reached. The summary of literature search is detailed in the PRISMA flow chart (Fig. 1).

### Summary

- Corneal opacity represents the 5th leading cause of blindness globally, with infectious keratitis (IK) being the main culprit.
- IK can be caused by a wide variety of pathogens, including bacteria, fungi, viruses, parasites and polymicrobial infection.
- Contact lens wear, trauma and ocular surface diseases are the three most common risk factors of IK.
- Several studies have highlighted the emerging trends in antimicrobial resistance in ocular infections, particularly in the US, China and India.
