# Correction to: Infectious keratitis: an update on epidemiology, causative microorganisms, risk factors, and antimicrobial resistance

**DOI:** 10.1038/s41433-021-01568-0

**Published:** 2021-05-11

**Authors:** Darren Shu Jeng Ting, Charlotte Shan Ho, Rashmi Deshmukh, Dalia G. Said, Harminder S. Dua

**Affiliations:** 1 Academic Ophthalmology, Division of Clinical Neuroscience, School of Medicine, University of Nottingham, Nottingham, UK; 2 Department of Ophthalmology, Queen’s Medical Centre, Nottingham, UK

**Keywords:** Corneal diseases, Epidemiology, Risk factors

Correction to: *Eye* (2021) **35**:1084–1101; 10.1038/s41433-020-01339-3; Article published online 21 January 2021

The article “Infectious keratitis: an update on epidemiology, causative microorganisms, risk factors, and antimicrobial resistance”, written by DSJT, CSH, RD, DGS, and HSD, was originally published Online First without Open Access. After publication in volume 35, issue 4, page 1084–1101 the author decided to opt for Open Choice and to make the article an Open Access publication. Therefore, the copyright of the article has been changed to © Author(s) 2021 and the article is forthwith distributed under the terms of the Creative Commons Attribution 4.0 International License, which permits use, sharing, adaptation, distribution and reproduction in any medium or format, as long as you give appropriate credit to the original author(s) and the source, provide a link to the Creative Commons license, and indicate if changes were made. The images or other third party material in this article are included in the article’s Creative Commons license, unless indicated otherwise in a credit line to the material. If material is not included in the article’s Creative Commons license and your intended use is not permitted by statutory regulation or exceeds the permitted use, you will need to obtain permission directly from the copyright holder. To view a copy of this license, visit http://creativecommons.org/licenses/by/4.0/.

